# COVID-19 lockdown – who cares? The first lockdown from the perspective of relatives of people with severe mental illness

**DOI:** 10.1186/s12889-022-13458-5

**Published:** 2022-06-02

**Authors:** Erlend Mork, Sofie R. Aminoff, Elizabeth Ann Barrett, Carmen Simonsen, Wenche ten Velden Hegelstad, Trine Vik Lagerberg, Ingrid Melle, Kristin Lie Romm

**Affiliations:** 1 Early Intervention in Psychosis Advisory Unit for Southeast Norway (TIPS Sør-Øst), Division of Mental Health and Addiction, Oslo University Hospital, Nydalen, P.O. box 4956, 0424 Oslo, Norway; 2 Norwegian Centre for Mental Disorders Research (NORMENT), Institute of Clinical Medicine, University of Oslo, Nydalen, P.O. box 4956, 0424 Oslo, Norway; 3 TIPS Centre for Clinical Research in Psychosis, Stavanger University Hospital, 4011 Stavanger, Norway; 4 Institute of Social Studies, Faculty of Social Sciences, University of Stavanger, P.O. box 8600, 4036 Stavanger, Norway; 5 Norwegian Centre for Mental Disorders Research (NORMENT), Division of Mental Health and Addiction, Oslo University Hospital, Nydalen, PB 4956, 0424 Oslo, Norway

**Keywords:** Carers, Family, Bipolar affective disorders, Psychotic disorders, Service users, Suicide

## Abstract

**Background:**

Informal care is vital to many people with severe mental illness under normal circumstances. Little is known about how extraordinary circumstances affect relatives with a family member with mental illness. This study investigated the consequences of the first COVID-19 lockdown in Norway from the perspective of relatives of persons with psychotic- and/or bipolar disorders: What were the challenges and for whom?

**Method:**

Relatives were invited to complete an online survey shortly after the first lockdown was initiated. Both quantitative and qualitative data were collected concerning experiences of relatives’ own and their affected family members’ health and situation. Two hundred and seventy-nine relatives completed the survey, mostly mothers and partners.

**Results:**

One-third of the relatives reported considerable deterioration in their family members’ mental health, and a substantial minority worried about severe self-harm or suicide. Main themes in the qualitative analyses were “Isolation and its effects on mental health”, “Worrying about the pandemic and its consequences”, “Increased symptomatology” and “Suicide”. Being a relative during the lockdown put heavy strain on the relatives’ own health, in particular disturbance of sleep, concentration, and the ability to take care of others in the family. Relatives of family members with psychotic bipolar disorder, not currently in treatment, or living with their family experienced the situation especially challenging.

**Conclusions:**

Many relatives found the first lockdown hard for their family. Efforts to integrate relatives’ perspectives in health care and contingency plans under normal circumstances could potentially alleviate some of the extra burden experienced by families during extraordinary circumstances.

**Supplementary Information:**

The online version contains supplementary material available at 10.1186/s12889-022-13458-5.

## Background

As the Coronavirus disease-19 (COVID-19) developed into a pandemic, most governments across the world initiated measures to prevent the spread of COVID-19, including physical and social distancing, self-isolation, and wearing face masks [1]. Furthermore, mental health services were disrupted in many countries during the COVID-19 pandemic [2], including Norway [3]. There were concerns that the measures and the worries people may have about contracting the virus, could have an adverse effect on the mental health in the general population or in subgroups [4–6]. Indeed, systematic reviews have indicated increased mental health symptoms, depression and/or anxiety in the general population [7, 8], increased suicidal behaviour in young adults [2], exhaustion in health care workers [2], and increased mental health problems in people with pre-existing physical health conditions [7]. The World Health Organization (WHO) state in a recent scientific brief that the global prevalence of anxiety and depression may have increased by 25% in the first year of the COVID-19 pandemic. For most other subgroups or diagnoses, it’s too early to conclude or the findings are mixed [2]. Findings from studies investigating how the COVID-19 pandemic affected people with pre-existing severe mental illness (SMI, in this study defined as psychotic- and/or bipolar disorders) are mixed [9, 10]. One study reports no change in affective or psychotic symptoms, and increased wellbeing in people with SMI [11]. Another study of older adults with bipolar disorder found less symptoms during COVID-19 than before the COVID-19 outbreak [12]. However, many studies find higher levels of distress, sleep disturbances, anxiety, depression, alcohol use or subjective cognitive dysfunction in people with SMI compared to the general population or before the pandemic [13–19]. Increased self-harm and suicidal behaviour have also been reported [20].

It seems reasonable to infer that increased symptoms may impact quality of life and health of the patients’ relatives as well [21, 22]. Relatives are often important in the informal care and support for people with psychotic- and/or bipolar disorders [23, 24]. Even if many families and their affected family member manage their situation [25], some relatives of persons with psychotic- and/or bipolar disorders report being more socially isolated, having reduced quality of life, and being more prone to mental health conditions compared to relatives of persons with other disorders or matched community samples [26, 27]. Research has shown that involving families of people with psychotic- [28] and bipolar disorders [29, 30] in treatment has a positive influence on outcome. Interventions designed to improve the emotional atmosphere in the family have been shown to reduce relapse and readmission [15], although there are exceptions [31]. Furthermore, psychoeducation, support groups, and family interventions appear to reduce distress and improve quality of life for relatives of people with severe mental illness [32, 33]. In bipolar disorder, family focused therapy [34] has been developed based on the same principles as for psychotic disorders, including psychoeducation, communications skills, and problem-solving. This therapy has been shown to reduce the risk of both manic relapse and depression [35]. However, we know from previous research that family work has been particularly difficult to implement, with barriers on several organisational levels [36]. Hence, there are substantial variations in the attention relatives and carers receive from health care services.

Onwumere and colleagues [37] found that informal (unpaid) carers of people with different mental or physical conditions reported high caregiving burden and mood and sleep disturbances during the COVID-19 pandemic. Given the challenges families with a family member with psychotic- and/or bipolar disorders face under normal circumstances, we suspected that they could be particularly vulnerable during the pandemic. In a search of the scientific literature, we found only three studies reporting on how the COVID-19 pandemic has affected the relatives of people with a psychotic and/or bipolar disorder specifically [20, 38, 39]. Caqueo-Urízar and colleagues [38] found that relatives of people with schizophrenia in Chile reported a slight to moderate effect of the COVID-19 measures on areas of daily life such as income, health, concern, social life and employment status, but with large variation across respondents. Muruganandam and colleagues [20] reported in a study from India that 30% of relatives to people with SMI reported an increase in the burden of taking care of patients during the COVID-19 pandemic. Yasuma and colleagues [39] found an association between higher daily caregiver burden and more difficult care experiences, especially worries about who would care for their family member with schizophrenia if the relative became infected with COVID-19. These studies suggests that the pandemic imposes extra challenges on a substantial subgroup of relatives. However, there is a need for more detailed information on the nature or impact of a heavier burden, and for whom it might be particularly challenging. Furthermore, in times of crises, people tend to increase the support for each other, strengthening a sense of community, of “being-in-this-together”. These and other unknown characteristics of the situation may strengthen or challenge the ability to cope under these extraordinary circumstances. The lack of knowledge about relatives’ experiences during the COVID-19 pandemic and previous pandemics has given rise to a call for studies investigating how relatives and carers of people with severe mental illness are affected by a pandemic [40, 41].

### Aims

The aims of this study were thus to investigate the first national COVID-19 lockdown in Norway (initiated March 12^th^, 2020) from the perspective of relatives of a family member with psychotic- and/or bipolar disorders. First, we wanted to explore how the lockdown affected a) their family member’s mental health, situation and ability to comply with the infection prevention measures, and b) the relatives’ own health and situation. Secondly, we wanted to explore whether these experiences differed according to diagnostic group, previous experience with psychoeducation, treatment status, illness course, and housing situation. The overarching goal was to contribute to the knowledge base for planning support and services for families with a person with severe mental illness under extraordinary circumstances.

## Methods

### Participants, procedure, and setting

Relatives of people with psychotic and/or bipolar disorders in Norway were invited to participate in an online anonymous survey designed for the purposes of this study. The survey was distributed via Facebook and Instagram, newsletters and e-mails, national and regional networks (including networks for psychoeducational family work and mental health service-clinicians), user-organisations (including Norwegian Bipolar Association, Mental Health Norway and Mental Health Carers Norway), charities, and mental health stakeholders. This can be described as snowball sampling, a type of availability sampling to reach out widely to recruit people who are difficult to identify and reach. The survey was open for relatives between May 15^th^ and June 15^th^, 2020. In the following we refer to the respondents as ‘relatives’ and the person with psychotic and/or bipolar disorder as the ‘family member’.

The Norwegian Government declared a nationwide lockdown due to the COVID-19 pandemic from March 12^th^, 2020, to April 6^th^, 2020. During the lockdown all schools and kindergartens were closed, and people were encouraged to work from home if possible. Most mental health outpatient services, including private practices, were closed, except those services considered necessary to avoid serious exacerbation of mental illness or life-threatening behaviour. There were strict limitations to the access to mental health services, and general practitioners warned people against turning up at their office unless strictly necessary. There were local differences in the implementation of governmental regulations, as well as differences between health trusts regarding the availability of teletherapy equipment and implementation during this period. After April 6th, the restrictions were gradually lifted.

### Measurements

In the absence of validated questionnaires for the Norwegian population to assess perceptions and impacts of pandemics on relatives and their family members, an ad hoc survey was developed based on discussions in the team of authors. The survey questions were revised and expanded according to feedback from relatives, researchers, psychiatric nurses, psychiatrists and clinical psychologists caring for or working with people with SMI. The survey (Additional file 1, English translation) consisted of 48 questions with fixed and open-ended response alternatives whereof 32 questions were included in this study. The results of the remaining questions have been published elsewhere [3]. We included questions about demographics, relatives’ experiences of how the lockdown had affected their family members’ mental health, situation, and ability to comply with the infection prevention measures, and questions concerning the impact of the lockdown on their own health and situation. All quantitative questions concerning the family members and relatives’ own health and situation had four response alternatives: “no”, “some”, “much” or “very much”. Responses to the open-ended questions were included in the qualitative analyses.

### Ethics approval and consent to participate

The authors assert that all procedures contributing to this work comply with the ethical standards of the relevant national and institutional committees and in accordance with the declaration of Helsinki. We disclosed and discussed the survey with the Regional Comittees for Medical Research Ethics South East Norway. The committee did not regard the project as medical or health professional research as it involved no patient data, as understood by the law, and hence the project fell outside of the provisions of the Health Research Act. The data protection officer at Oslo University Hospital approved the project (case number: 20/09173). The responders were informed of the purpose of the study, possible advantages and disadvantages of responding, including that the survey included questions about negative consequences of the COVID-19 lockdown, and they were given information about which services to contact if they needed someone to talk to. Taking the survey after given the information was considered a consent. The survey was anonymous, so we do not know whether someone under the age of 18 completed the survey.

### Analyses

Statistical analyses of questions with fixed response alternatives were performed with IBM SPSS Statistics 26. In the text, we report results in percent (n) for everyone who answered affirmative to some extent (“some”, “much” and “very much”) for a specific question, if not otherwise stated. In the tables (Table 1 and 2) and figure the variables were dichotomized into “to a large extent” (much/very much) versus some/no. Group differences (diagnostic groups) were analysed using chi square tests with a pre-set significance level of 0.05. The significance level was Bonferroni corrected for multiple tests in the analyses of “Family members health and situation” to 0.00625 (0.05/8) and “Own health and situation” to 0.005 (0.05/10). Tests were two-sided.

**Table 1:** Sample characteristics, total and according to diagnostic group

		Total sample n = 279	Family member with			Statistics
			Bipolar disorder (BD) *n* = 135	Psychotic Bipolar disorder (PBD) *n* = 57	Psychotic disorder (PD) *n* = 87	
		n (%)	n (%)	n (%)	n (%)	X^2^ (p)
Relationship	Mother	121 (43)	43 (32)	26 (46)	52 (60)	X^2^ = 25.9 (0.001)^a^
	Father	9 (3)	4 (3)	3 (5)	2 (2)	
	Sibling	34 (12)	18 (13)	5 (9)	11 (13)	
	Spouse/partner	65 (23)	45 (33)	13 (23)	7 (8)	
	Other (offspring)	50 (18)	25 (19)	10 (18)	15 (17)	
≤ 1 year since family members’ first treatment		10 (4)	7 (5)	5 (9)	14 (16)	X^2^ = 7.5 (0.024)^a^
Affected family member currently in treatment	Yes	228 (80)	105 (78)	41 (72)	78 (90)	X^2^ = 7.9 (0.019)^a^
Received psychoeducation as a relative	Yes	80 (29)	34 (25)	18 (32)	28 (32)	X^2^ = 1.6 (0.458)
Living in same household	Yes	107 (39)	57 (42)	23 (40)	27 (31)	X^2^ = 2.9 (0.232)

^a^Significant results

**Table 2:** Relatives experience of their family members and own health and situation

	Total sample	Family member with			Statistics X^2^ (p)
	*N* = 279	Bipolar disorder *n* = 135, 48%	Psychotic Bipolar disorder *n* = 57, 20%	Psychotic disorder *n* = 87, 31%	
	To a large extent	To a large extent	To a large extent	To a large extent	
	n (%)	n (%)	n (%)	n (%)	
Family members health and situation					
Mental health improved during the pandemic?	22 (8)	15 (11)	5 (9)	2 (2)	X^2^ = 5.7 (0.057)
-If improved, improved because of pandemic?	8 (3)	7 (5)	1 (2)	1(0)	X^2^ = 5.4 (0.066)
Mental health deteriorated during the pandemic?	93 (33)	38 (28)	25 (44)	30 (35)	X^2^ = 4.5 (0.104)
-If deteriorated, deteriorated because of pandemic?	69 (25)	27 (20)	18 (32)	24 (28)	X^2^ = 3.4 (0.179)
Worries of danger to self or other family members due to difficulties following infection prevention measures?	35 (13)	7 (5)	17 (30)	11 (13)	X^2^ = 22.2 (< 0.001)^a^
Worries of severe self-harm or suicide as a consequence of pandemic?	54 (19)	22 (16)	16 (28)	16 (18)	X^2^ = 3.6 (0.162)
Worries of acting out or violence as a consequence of pandemic?	33 (12)	5 (4)	15 (26)	13 (15)	X^2^ = 20.8 (< 0.001)^a^
Worries of not getting help from mental health services in case of deterioration of illness?	141 (51)	61 (45)	36 (63)	44 (51)	X^2^ = 5.2 (0.075)
Own health and situation					
Has your responsibility as caretaker increased during the pandemic?	117 (42)	49 (36)	33 (58)	35 (40)	X^2^ = 7.8 (0.020)
Has the pandemic had negative consequences for your economy?	32 (12)	18 (13)	9 (16)	5 (6)	X^2^ = 4.3 (0.116)
Have you feared being infected by the virus and not being able to take care of your family member?	62 (22)	22 (16)	19 (33)	21 (24)	X^2^ = 7.0 (0.030)
Have you felt personally responsible for your family members’ ability to abide with the infection prevention measures?	56 (20)	18 (13)	18 (32)	20 (23)	X^2^ = 9.0 (0.011)
Have concerns related to your family members’ health during the pandemic affected your					
-sleep?	79 (28)	21 (16)	28 (49)	30 (35)	X^2^ = 24.6 (< 0.001)^a^
-appetite?	29 (10)	7 (5)	13 (23)	9 (10)	X^2^ = 13.3 (0.001)^a^
-concentration?	81 (29)	31 (23)	25 (44)	25 (29)	X^2^ = 8.5 (0.014)
-ability to take care of yourself?	29 (10)	8 (6)	11 (19)	10 (12)	X^2^ = 7.9 (0.020)
-ability to take care of other family members?	33 (12)	4 (3)	16 (28)	13 (15)	X^2^ = 25.4 (< 0.001)^a^
Has the role of relative affected your ability to perform your tasks at work during this period?	53 (19)	20 (15)	15 (26)	18 (21)	X^2^ = 3.7 (0.159)

^a^Significant results

Qualitative data were analysed according to the principles of systematic text condensation which is a descriptive and explorative method for thematic cross-case analyses of different types of data, including written texts [42]. Analysis was conducted according to this qualitative study approach in four steps: (1) EM, KLR and SRA read all the answers to the open-ended questions to achieve an overall impression and to look for preliminary themes related to the aims. (2) The text was broken down into manageable meaning units and organized into code groups. (3) We condensed the meaning under each code group. (4) Lastly, we developed an analytic text, a synthesis, of each category relevant for the study. The transcripts were reviewed three or more times by EM, KLR and SRA to ensure accurate representation and interpretation. Any disagreement in each step were dissolved by consensus discussions. All designated themes and categories were compared with the original survey answers, and appropriate quotations were selected. We used NVivo (version 10; QSR International LLC) for step 2 and step 3 of the qualitative data analysis.

### Sample characteristics

The total sample consisted of 279 relatives of whom 48% (n = 135) defined themselves as relatives of a family member with bipolar disorder, 31% (n = 87) as relatives of a family member with psychosis, and 20% (n = 57) replied affirmative to both psychosis and bipolar disorder. Based on a review of the answers to the open-ended questions, we termed this latter group “Psychotic bipolar disorder”. However, we cannot rule out that a few relatives in the Psychotic bipolar group had two different family members (one with psychoses and one with bipolar disorder) or that some in the bipolar disorder group also had had psychotic episodes. Table 1 shows the characteristics of the relatives according to diagnostic group. Relatives of persons with psychosis were significantly more often mothers, and their affected family member were more often currently in treatment and in their first year of treatment. Relatives from all five Health Regions of Norway were included in the sample, with fairly good representativity based on population proportions (data not shown).

## Results

### The family member's health and situation

#### Mental health

##### Quantitative

Twenty-seven percent (*n* = 74) reported at least some improvement in the mental health of their family member during the lockdown, while 71% (*n* = 198) reported perceiving that their family member’s mental health deteriorated during the lockdown. Table 2 details that 33% (*n* = 93) reported perceiving deterioration to a large extent in their family member. A majority of relatives, 85% (*n* = 237) worried about not getting help from mental health services if their family member should deteriorate during lockdown. Interestingly, among relatives who reported having received some form of mental health psychoeducation for families, significantly fewer reported worry about not getting help from mental health services (psychoeducation: 75% (*n* = 60) vs no psychoeducation: 88% (*n* = 176), X^2^ = 7,9, df = 1, *p* = 0.005).

##### Qualitative

A larger proportion of relatives provided open responses regarding deterioration (64%, *n* = 179) than improvement (22%, n = 39). From the qualitative analysis of deterioration-responses, we subtracted four themes: “Isolation and its effects on mental health”, “Worrying about the pandemic and its consequences”, “Increased symptomatology” and “Suicide” (Table 3 presents details and quote examples). Descriptions of the affected family member isolating or experiencing involuntary isolation due to the lockdown was highly prevalent. Many relatives found it harder than usual to reach out to their family member. Descriptions of increased core- and comorbid symptoms, increased worry and loneliness, and increased craving of social contact were attributed to the isolation posed by fears of infection and/or social distancing. One relative stated: *"Loneliness increases psychosis risk, (it is) difficult to do the right thing, be calm; avoid getting scared, exhausted and anxious."*

**Table 3:** Questions and themes with exemplifying quotes

Question (Q)	Themes	Quote
**IMPROVED** **Q: Has the mental health of your family member improved during the pandemic?** If improvement, feel free to elaborate how the pandemic has had a positive effect	More tranquillity	*That my brother copes with the corona restrictions so well, and both notices and appreciates that everyone around him also lives a quieter life, has more time and gives more contact. Everyone in the family is happy about this, which has also given us a new perspective on how much this means to a sensitive and intermittently introverted person. (sibling)*
	More time – more in touch	*When the school turned into home school, we saw great change. Less unattainable demands and more sense of mastery. Less social difficulties and more help with regulation. (mother)*
	More like others	*Does not feel so "alone". All people need to be a lot at home/inside and she is more in line with society in this time. (mother)*
**DETERIORATED** **Q: Has the mental health of your family member deteriorated during the crisis?** If deteriorated, elaborate how the pandemic has had a negative impact on your family member	Isolation and its effect on mental health	*My mother needs contact with children and grandchildren to stay away from depression. Without this contact, she became depressed and got a lot of anxiety, as well as became easily paranoid. (offspring)*
	Worrying about the pandemic and its consequences	*Mom loses perception of reality more easily. Concerns that have previously led to psychoses, about surveillance, disease and the future come more than before. (offspring)*
	Increased symptomatology	*Being quarantined was the trigger that caused my spouse to go into a powerful psychosis. (spouse)*
	Suicide	*She was laid off from the part-time job … and missed the job. For a while I kept her going with a … project and she was embarking on a couple of other projects. Then all of a sudden, she took her own life. She was depressed, but has always rejected any question of suicide before, so we don't know what's happened. But I'm sure the corona crisis had some part in it. (mother)*
**Own health** Q: Are there other things you would like to share with us that are important regarding your role as relative in this situation?	Strain on own health	*At times demanding to be supportive, to be present all the time. To sleep together, to calm down, and talk for much of the day. …the same is expressed all the time. Must be thoughtful answers. Must be patient and understanding. Even if you are very tired. (mother)*

From the relatives’ descriptions of improvement, we subtracted three main themes: “More tranquillity”, “More frequent contact” and “Feeling more like others” (Table 3). Several relatives reported that the reduced pressure to participate in society during the lockdown made the whole family become more relaxed. They communicated better, and the frequency of contact with their family members increased. Relatives attributed this to having more time available, and increased use of digital communication tools made it possible. One relative stated that: *"Due to this crisis, we came back to each other, after almost two years without any contact whatsoever”.*

#### Fear of destructive or dangerous behaviour

##### Quantitative

**Fig. 1:**
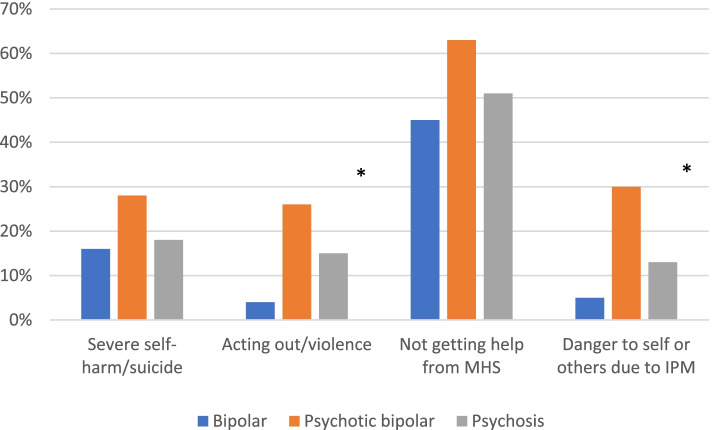
Percentage of relatives worrying ‘to a large extent’ about destructive or dangerous outcomes as a consequence of the pandemic. *Significant group differences. For statistics and group sizes, see Table 2 for details. MHS = Mental health services, IPM = Infection prevention measures

Thirty-four percent (*n* = 94) of relatives feared their affected family member would pose a threat to self or others due to difficulties in complying with the infection prevention measures. Thirty-six percent (*n* = 74) had *some* fear that their family member would be acting out or become violent. The proportion worrying about severe self-harm or suicide was somewhat higher (43%, *n* = 139) and a substantial minority of 19% (*n* = 54) to a large extent feared suicide or severe self-harm in their family member. Figure 1 shows the proportion reporting fear of destructive or dangerous behaviour according to diagnostic group. More than 25% in the psychotic bipolar disorder group reported such fears on all four measures detailed in Fig. 1, with significant differences between diagnostic groups on two measures: Fear that their family member would pose a threat to self or others due to difficulties in complying with the infection prevention measures, and Fear of acting out or violence as a consequence of the pandemic (Fig. 1, Table 2). Relatives of family members with psychotic bipolar disorder group reported these fears most often.

##### Qualitative

Several relatives described increased suicidal behaviour in their family member, which they associated with either increased isolation or increased symptoms in their family members. Of note, three relatives reported that their family member died in suicide during the first lockdown.

### Relatives’ own health and situation during the crises

#### Quantitative

**Fig. 2:**
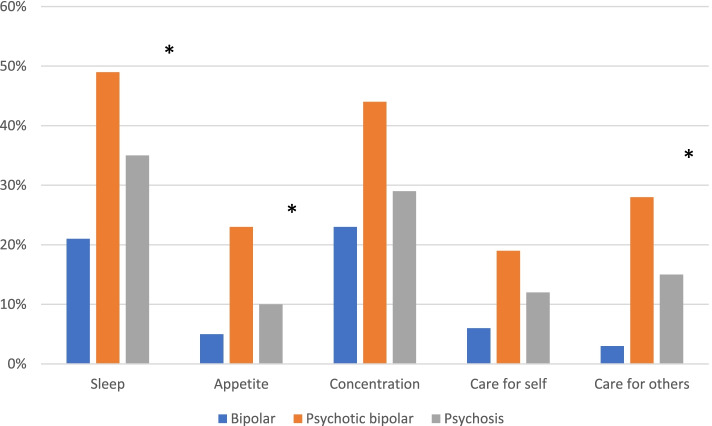
Have worries concerning your family members’ health during the pandemic affected your sleep/appetite/concentration/care for self/care for others? Percentage replying ‘to a large extent’ according to group.*Significant group differences, see Table 2

Many relatives reported managing well through the lockdown in terms of their economy, ability to work, and to care for themselves and the family (Table 2). However, 83% (n = 231) felt that the pandemic increased their responsibility as relatives. Furthermore, worries concerning their family members’ health during the pandemic affected their sleep (62%, *n* = 173), concentration (71%, n = 199), and ability to care for other family members (39%, *n* = 110). Figure 2 shows the proportion reporting worries according to diagnostic group. Almost half the relatives in the psychotic bipolar disorder group and 35% in the psychosis group reported that such worries affected their sleep to a large extent (Table 2).

The degree to which they reported sleep to be affected by the health of their family member correlated with the degree to which they feared severe self-harm or suicide in their family member (r_s_ = 0.56, *p* < 0.001); worried about not getting help from mental health services in case of deterioration (r_s_ = 0.52, *p* < 0.001); observed deterioration in their family member’s mental health (r_s_ = 0.48, *p* < 0.001); feared acting out or violence (r_s_ = 0.47, *p* < 0.001); felt increased responsibility as caretaker (r_s_ = 0.44, *p* < 0.001); feared that their family member would pose a threat to self or others due to difficulties in complying with the infection prevention measures (r_s_ = 0.26, *p* < 0.001); felt personally responsible for their family members’ ability to comply with the infection prevention measures (r_s_ = 0.23, *p* < 0.001); felt the pandemic had negative consequences for their economy (r_s_ = 0.21, *p* = 0.001); and negatively correlated with the degree to which they observed improvement their family member’s mental health (r_s_ = -0.20, *p* = 0.001).

#### Qualitative

Thirty-two percent (*n* = 90) gave written responses to the question: “Is there anything else you want to share with us concerning the role as a relative in this situation?” (Table 3). The responses revealed that many relatives felt extremely exhausted. Some described that it was particularly challenging living so close together, with no time off on their own and hardly any relief. Others commented that their threshold for losing their temper and patience was markedly lower than usual. This period caused additional worries and tension, and relatives reported feeling unsafe and scared. Several described that their family “was falling apart”.

#### Relatives with family member not currently in treatment

The relatives with family members currently not in treatment differed from other relatives on three parameters with potential strong impact on their quality of life: They reported significantly more often that the lockdown *to a large extent* influenced their ability to take care of themselves (n = 12/55, 22% vs n = 17/224 (8%), X^2^ = 9,6, df = 1, *p* = 0.002.), that the lockdown negatively influenced their economy (not in treatment: n = 14 (26%) vs in treatment: *n* = 18 (8%), X^2^ = 13,2, df = 1, *p* < 0.001), and that they more often feared violence or acting out from their family member (*n* = 11 (20%) vs *n* = 22 (10%), X^2^ = 4,4, df = 1, *p* = 0.04).

#### Relatives of family members in first year of treatment

The relatives with family members who had started their first treatment within the last year where similar to other relatives on all except one measure: They more often reported that being a relative *to a large extent* had affected their ability to perform tasks at work during the lockdown (< = 1 year: n = 11/26, 42%, vs > 1 year: n = 42/253, 17%, X^2^ = 10,1, df = 1, *p* = 0.001).

#### Relatives living in the same household as their family member

Relatives living in the same household as their family member more often reported that the lockdown *to a large extent* influenced their ability to take care of themselves compared to relatives not living with their family member (*n* = 17/107, 16% vs *n* = 12/172, 7%, X^2^ = 5,6, df = 1, *p* = 0.018) and that being a relative *to a large extent* had affected their ability to perform tasks at work during the lockdown negatively (*n* = 65/107, 70%, vs *n* = 75/172, 44%, X^2^ = 7,8, df = 1, *p* = 0.005).

## Discussion

The main findings in this study are that although many relatives reported that their family member managed the first COVID-19 lockdown well, one third observed marked deterioration in their family member. Second, being a relative during the lockdown put heavy strain on the relatives’ own health, in particular disturbance of sleep, concentration, and the ability to take care of others in the family. Third, relatives of family members with psychotic bipolar disorder experienced the situation especially challenging, as did relatives of family members not currently in treatment, relatives of family members in first treatment, and families living together.

It is worth noticing the wide range of experiences reported in this study, as in the studies from a Chilean [38] and Japanese [39] context. Relatives reporting the family managing the lockdown well in our study, related this to the slowing down of society, which brought more tranquillity, more contact and even reunion for some families. This notwithstanding, most relatives (85%) worried that their family member would not get help from mental health services if needed, in line with the finding from Yasuma and colleagues from Japan [39]. Relatives in our study who had received psychoeducation at some point worried less about service availability. This suggests that systematic psychoeducation to families during normal circumstances is helpful for alleviating such concerns. One possible explanation for this finding, is that trust in mental health services increases, as relatives are engaged in the services and/or increase their knowledge. Another explanation might be that family psychoeducation professionals, who had on-going working relationships with the families, remained available for contact by phone or video chat during the lockdown. We found that relatives of family members not in treatment were more often reporting that worries about their family members health affected their ability to take care of themselves, had a negative influence on their economy, and that they more often worried about acting out or violence. This suggests that offering relatives psychoeducation and support, even when their family member is not in treatment, may be important, especially during a crisis.

### Relatives of family members with psychotic bipolar disorder

Our findings suggest that relatives of people with psychotic bipolar disorder carry a particularly heavy burden. There are previous reports of both higher [43, 44] and lower [45] carer burden for family members with bipolar disorder compared to those with schizophrenia. Taking into consideration the heterogeneity of these disorders, psychiatric diagnoses may be of limited value in understanding the caregiver burden, and that it may be more informative to focus on specific symptoms or challenging behaviours [46]. We found a strong association between the extent to which the relatives reported their sleep to be affected and fears of self-destructive, dangerous or aggressive behaviours in their affected family member. This is in line with the notion that such behaviours may have adverse effects on relatives, regardless of diagnoses. Previous studies have found that manic episodes are particularly burdensome to relatives [46–48], perhaps related to characteristics such as aggressiveness, lack of insight, and financial problems [47, 49, 50]. Dore [49] also found that suicidal behaviour, especially in depressive episodes were burdensome to relatives. Similarly, reports of patient-initiated violence in people with psychosis have previously been found to be associated with poorer wellbeing and more negative appraisal of caregiving in relatives [51, 52]. Such a “double jeopardy”; being exposed both to affective and psychotic episodes, might be one possible explanation for the particularly high proportion of relatives worrying about violence/acting out and other dangerous or self-destructive behaviours in the psychotic bipolar group in our sample.

### The relative’s own health and situation

The high proportion of relatives reporting that worries concerning their family members’ health affected their sleep, is a cause for concern, and in line with Onwumere and colleagues finding among informal caregivers in general [37]. A recent study investigating sleep disturbance in early psychosis caregivers found it to be associated with higher levels of distress and with negative appraisals about caregiving [53]. In our material, many relatives described feeling extremely exhausted or tired, and several commented that the threshold for losing their temper and patience were markedly lower than usual, describing situations with few opportunities for rest or time of their own. This suggests that some relatives were experiencing a vicious circle during the first lockdown: deterioration in their affected family member, worries about destructive behaviours, feelings of entrapment and increasing exhaustion and disturbances of sleep. Breaking such circles, e.g. by making sleep interventions available or teaching skills in managing stress and/or difficult situations, may be important to support both the relatives and the family as a whole.

### Strengths and limitations

The strength of this study is that it had nationwide participation – with good regional representativity. Furthermore, we reached relatives with family members currently not in treatment. They represent families that are often hard to reach in research, and the findings from this study suggest that some of these families may carry heavy burdens. The deliberate choice of not asking the relatives to identify themselves may have increased the response rate in this group. Nevertheless, the lack of more detailed demographic information is a limitation. We considered the relationship to their family member as the most important information, as studies have shown that the majority of informal carers are the parents (usually mothers), followed by spouses and siblings [54]. The use of social media in the distribution may have contributed to fewer fathers responding to the survey. Although gender differences in informal care (more women) have been reported [55], the differences seem to have decreased over time [56]. Thus, reaching a larger group of fathers and knowing more about gender and age could have painted a more detailed picture of relatives’ experiences. The lack of comparison group and longitudinal data prohibit inferences about differences with relatives in the general population. The authors who did the qualitative analysis (KLR, SRA, EM) are involved in implementation of family- and relatives focused interventions for people with severe mental illness. Pre-understanding related to these experiences may have influenced the qualitative analyses. In order to mitigate such effects, we strived to balance the questions, addressing both advantages and disadvantages of the situation, and to follow the qualitative analysis method strictly. Another limitation is that availability sampling and self-selection was used to recruit participants to the survey. Because of this, there is no way to determine whether the respondents are representative of relatives in the general population of interest. Therefore, there are limitations in the generalizability of the results. Also, at the time of the study, there were no validated questionnaires available in Norwegian to assess the perceived impact of the COVID-19 pandemic from relatives’ perspective, so a survey was developed for the purposes of this study. Furthermore, the survey was conducted under extraordinary circumstances, in a high-income country with public health care system. Lastly, we did not determine the sample size before the study. For these reasons, the findings should be seen as hypotheses generating and interpreted and generalized to other contexts and situations with caution.

### Involving relatives of family members with psychotic- and bipolar disorders in health care

Family work has not been implemented on an adequate scale for people with psychotic and/or bipolar disorders, as reflected in the current study. Relatives who had received this type of support experienced less fear of not getting help from mental health services compared to those who did not. Most families still lack the tools necessary to provide sufficient support, and our study suggests that the pandemic has had a negative impact on a large proportion of both patients and relatives in terms of mental health and quality of life. Relatives are often both willing and able to step up during a crisis; they feel responsible for doing so. However, they are rarely supported and consulted as part of any contingency plan. As noted in a comment in relation to the current pandemic, the health-care community cannot afford to lose the aid of caregivers [41]. To enable families to take care of themselves and their family members, they need education, tools and direct support from mental health services. Health services are high-risk organisations, and as such, they should prepare for possible scenarios beyond major accidents and terrorist attacks. Our results lead us to reflect on three issues related to preparedness for future extraordinary events: 1) We need contingency plans that include scenarios with reduced provision of care over time; 2) We need to acknowledge relatives as an integrated part of the care for family members with a severe mental illness, especially during a long term crisis, and 3) A good working alliance with relatives has to be established as early as possible, to prepare for support during crisis. Systematic efforts to make family interventions [29, 30] and other interventions aimed at improving the experience of caring for people with severe mental illness [32, 33] widely available, would be an important first step towards such a goal. As new digital solutions for relatives of people with severe mental disorders open up for new approaches [57, 58], there is a need for more research on how we can apply these possibilities for education, communication and collaboration to enhance good patient and family outcome and be better prepared in the future. Chances are it will be needed.

## Supplementary Information

**Additional file 1.** National COVID-19 relatives survey.

## Data Availability

Some respondents shared sensitive stories. Out of respect for the respondents, we do not wish to share our raw data publicly since it was not stated in the informed consent that raw data would be published. The data used and/or analysed that support the findings of this study are available from the corresponding author, EM, upon reasonable request.

## Abbreviations

COVID-19: Coronavirus Disease of 2019
BD: Bipolar Disorder
PBD: Psychotic Bipolar Disorder
PD: Psychotic Disorder
MHS: Mental Health Services
IPM: Infection Prevention Measures
SMI: Severe Mental Illness
