# Supplementary material for: COVID-19 lockdown – who cares? The first lockdown from the perspective of relatives of people with severe mental illness
Source: BMC Public Health. 2022 Jun 2;22:1104. doi: 10.1186/s12889-022-13458-5 (PMC9162484; doi:10.1186/s12889-022-13458-5)
Supplement: Supplementary file 1 — Additional file 1. National COVID-19 relatives survey. [file 12889_2022_13458_MOESM1_ESM.pdf]

National COVID-19 relatives survey

Translated from Norwegian to English

How has the COVID-19 pandemic outbreak affected you as a relative of someone with a severe mental illness?

The aim of the project and why you are being asked to participate

The COVID-19 pandemic has led to major changes for all of us. We are asking if you would like to participate in a survey. Any person who is a relative to someone with psychosis or bipolar disorder may participate. The aim of the study is to learn more about how the COVID-19 outbreak has affected the situation for the relatives of a person with psychosis or bipolar disorder.

What does participation involve?

We will ask you to answer some questions about how the COVID-19 outbreak has affected your role as a relative in everyday life: mentally, financially and in the relationship with your family member. It will take about 5–10 minutes to fill out the survey. The survey is anonymous. Therefore, there will be no personal follow-up given to you based on the survey.

Possible advantages and disadvantages

The possible disadvantages of participating is the time you spend filling out the questionnaire and that the survey may cause an increased focus on concerns. One possible benefit is that it may help you think through your situation and see if you can make any changes. Your contribution may provide valuable information on how health services should be designed for similar situations in the future. If you need someone to talk to, we ask you contact your general practitioner/therapist, your local community mental health service or a helpline.

The project is approved by the Data Protection Officer at Oslo University Hospital and Project Manager Kristin Lie Romm is responsible for the data and privacy protection of the project. If you have any questions about the survey, contact project manager Kristin Lie Romm through email at k.l.romm@medisin.uio.no. It is not possible to withdraw from the survey after submitting your form. The project is managed by The Early Intervention in Psychosis Advisory Unit for Southeast Norway at Oslo University Hospital, in collaboration with TIPS Stavanger, Stavanger University Hospital and The Norwegian Centre for Mental Disorders Research, NORMENT, Oslo University Hospital and University of Oslo.

Think about the period since March 13, 2020, when Norway shut down much of its activity because of the COVID-19 crisis. The term family member is used in this survey to describe the person you are a relative to.

Questions that are relevant to this manuscript are highlighted in blue. The ones that are black are the subject of another paper.

I am a relative to a person with psychosis	Yes	No			
I am a relative to a person with bipolar disorder	Yes	No			
What is your relationship to your family member	Mother	Father	Sibling	Partner	Other
Which regional health trust does your family member belong to?	North	Middle	West	South-East	
Which regional health trust do you yourself belong to?	North	Middle	West	South-East	
How long have you been a relative to someone with a psychosis or bipolar disorder (measured form the time the person received treatment for the first time)	< 6 months	6 months - 1 year	1-2 years	3 years or more	
Have you ever participated in psychoeducational family group, bipolar course or something similar as a relative?	Yes	*No*			
Does your family member normally live in the same household as you?	Yes	No			
If no, does your family member live in the same household as you during the COVID-19 crisis?	All the time	Often	Part of the time	Seldom	Not at all
1. Do you feel that the COVID-19 crisis has affected you in your role as a relative?	No	Some	Much	Very much	
2. Do you feel that your responsibility as a caregiver has increased in connection with the COVID-19 crisis?	No	Some	Much	Very much	
3. Has your family member's mental health improved during the crisis?	No	Some	Much	Very much	
3b. - <if improved - do you think the improvement is a consequence of the crisis?	No	Some	Much	Very much	
3c. If improved, please elaborate how the crisis has had a positive effect	Open ended question with written responses				
4. Did the mental health of your family member deteriorate during the crisis?	No	Some	Much	Very much	
4b. – If yes, do you think the deterioration is a consequence of the crisis?	No	Some	Much	Very much	
If deteriorated, can you tell us how the crisis has affected your family member in a negative way?	Open ended question with written responses				
6. Have you experienced that it has been difficult for your family member to adhere to the infection prevention measures of the health authorities?	No	Some	Much	Very much	
7. Have you felt a personal responsibility that your family member adheres to the infection-prevention measures?	No	Some	Much	Very much	
8. If you have been concerned about compliance with the infection-prevention measures, have you been afraid that your family member will pose a danger to themselves or other family members?	No	Some	Much	Very much	
9. Have you been worried to be infected yourself so that you could not take care of your family member?	No	some	Much	Very much	
10. Have you been worried that your family member would be infected with COVID-19 and manage the					

situation worse than the average person because of complications related to					
- physical health?	No	Some	Much	Very much	
- mental health?	No	Some	Much	Very much	
- medication?	No	Some	Much	Very much	
12. Has the COVID-19 crisis negatively affected your economy?	No	Some	Much	Very much	
12 Has the role of relative affected your ability to perform your tasks at work during this period?	No	Some	Much	Very much	
13. Has the atmosphere/communication in your family been better than normal during this period?	No	Some	Much	Very much	
-If better, does it have anything to do with the situation of your family member?	No	Some	Much	Very much	
Please provide more information if you would like to	Open ended question with written responses				
14. Has the atmosphere/communication in your family been worse than normal during this period?	No	Some	Much	Very much	
14b. – If worse, does it have anything to do with the situation of your family member?	No	Some	Much	Very much	
Please provide more information if you like to	Open ended question with written responses				
15. Have you been worried that your family member will take his/her own life or seriously harm him/herself as a consequence of the COVID-19 crisis?	No	Some	Much	Very much	
16. Have you been worried that your family member will become violent or act out as a consequence of the COVID-19 crisis?	No	Some	Much	Very much	
17. Have you been worried that your family member would suffer a deterioration in his/her illness during the COVID-19 crisis but not get help from the mental health care services during the crisis?	No	Some	Much	Very much	
18. Have concerns related to the health of your family member during the COVID-19 crisis affected:					
-your sleep?	No	Some	Much	Very much	
-your appetite?	No	Some	Much	Very much	
-your concentration?	No	Some	Much	Very much	
-your ability to take care of yourselves?	No	Some	Much	Very much	
-your ability to care of others in the family?	No	Some	Much	Very much	
19. Has the treatment offered to your family member been reduced during the crisis?	No	Some	Much	Very much	Not in treatment
20. Have you received satisfactory information about the treatment of your family member during the crisis?	No	Some	Much	Very much	Not in treatment
20. Has your family member received reduced services from the municipality during this period?	No	Some	Much	Very much	Do not use municipality services
21 Have you used any of the following opportunities to get support during the crisis? ? (tick off multiple answers, if needed)<br>*-Web-based information outside of hospital*<br>*-Hospital homepages (hospital, District Psychiatric Centres (DPS), outpatient clinics)*<br>*- Face-to-face consultation with health care professional (hospitals, DPS, outpatient clinics)*<br>*- Video consultation with health care professional (hospitals, DPS, outpatient clinics)*<br>*- Phone consultation with health care professional (hospitals, DPS, outpatient clinics)*<br>*-Chat function (hospitals, DPS, outpatient clinics)*	☐<br>☐<br>☐<br>☐<br>☐<br>☐				

Not applicable	☐				
If so, what helped you the most?	Open ended question with written responses				
What would you wish the health care services would prioritize higher regarding relatives during a crisis like this?<br>*(tick of multiple answers, if needed)*<br>*Web-based information*<br>*Better hospital webpages (hospital, DPS, Outpatient clinics)*<br>*Offer face-to-face consultations*<br>*Offer video consultation*<br>*Offer phone consultation*<br>*Offer Chat*<br>*Not applicable*	 <br><br><br>☐<br>☐<br><br>☐<br>☐<br>☐<br>☐<br>☐				
Please elaborate	Open ended question with written responses				
Are there other things you would like to share with us that you think are important in relation to your role as a relative in this situation?	Open ended question with written responses				

Thank you for your contribution!
